# Assessing the construct validity of a theory of mind battery adapted to Tunisian school-aged children

**DOI:** 10.3389/fpsyt.2023.974174

**Published:** 2023-03-09

**Authors:** Imène Soumaya Salhi, Céline Lancelot, Yousri Marzouki, Wided Souissi, Aya Nejiba Besbes, Didier Le Gall, Tarek Bellaj

**Affiliations:** ^1^Tunis University, Department of Psychology, Faculty of Humanities at Tunis, Tunis, Tunisia; ^2^Laboratoire de Psychologie des Pays de la Loire (LPPL), Université d’Angers, Angers, France; ^3^Psychology Program, Department of Social Sciences, College of Arts and Sciences, Qatar University, Doha, Qatar; ^4^Laboratoire de Psychologie des Pays de la Loire (LPPL), Centre Hospitalier Universitaire (CHU) d’Angers, Université d’Angers, Angers, France

**Keywords:** theory of mind, psychological assessment, test adaptation, construct validity, school-aged children

## Abstract

**Background:**

Theory of mind (ToM) refers to the ability to understand others’ states of mind, desires, emotions, beliefs, and intentions to predict the content of their mental representations. Two major dimensions within ToM have been studied. The first is the type of inferred mental state, which can be cognitive or affective. The second comprises the types of processes involved according to their degree of complexity (first- and second-order false belief and advanced ToM). ToM acquisition is fundamental—a key component in the development of everyday human social interactions. ToM deficits have been reported in various neurodevelopmental disorders through various tools assessing disparate facets of social cognition. Nevertheless, Tunisian practitioners and researchers lack a linguistically and culturally appropriate psychometric tool for ToM assessment among school-aged children.

**Objective:**

To assess the construct validity of a translated and adapted French ToM Battery for Arabic-speaking Tunisian school-aged children.

**Methods:**

The focal ToM Battery was designed with neuropsychological and neurodevelopmental theory and composed of 10 subtests distributed evenly in three parts: Pre-conceptual, cognitive, and affective ToM. Translated and adapted to the Tunisian sociocultural context, this ToM battery was individually administered to 179 neurotypical Tunisian children (90 girls and 89 boys) aged 7–12 years.

**Results:**

After controlling for the age effect, construct validity was empirically confirmed on two dimensions (cognitive and affective) *via* structural equation modeling (SEM) analysis, demonstrating that this solution has a good fit. The results confirmed that the age affected differentially the performance obtained on ToM tasks based on the two components of the battery.

**Conclusion:**

Our findings confirm that the Tunisian version of the ToM Battery has robust construct validity for the assessment of cognitive and affective ToM in Tunisian school-aged children; hence, it could be adopted in clinical and research settings.

## Introduction

Social cognition is a vast conceptual field that brings together all the mental processes that make it possible to perceive and process social stimuli and signals, such as the recognition of emotions, empathy, moral judgment, and theory of mind (ToM). Therefore, it is crucial to develop and adapt tools for evaluating these processes in research and clinical activities, e.g., the psychological examination of the developmental aspects of these sociocognitive skills in children. In addition, in child clinics, psychiatric pathologies have recently garnered interest in the field of social cognitive neuroscience because they are characterized by both cognitive disorders and relational and affective disorders that can be linked to ToM dysfunction. One psychiatric pathology that has been the subject of extensive work in recent decades is autism spectrum disorder (ASD). Through the conjunction of affective and cognitive disorders it manifests, ASD functions as a privileged experimental paradigm for interrogating the relationship between affective and cognitive mechanisms and, specifically, their intricacies during development (1). Indeed, seminal experiments involving this pediatric population have reported ToM deficits in preschool-aged children with ASD *via* various paradigms adapted to their mental age and explorations of various mental states. These deficits manifest in the inability of children with ASD to distinguish physical from mental events, appearance, and reality (2–4) or to understand that emotions can be linked to complex mental states, such as beliefs (1, 5–8). Consequently, the ToM dysfunctions observed among the latter correspond with the main communication and social interaction disorders as well as salient and frequently noted symbolization difficulties (9). This therefore explains the rationale for clinicians possessing appropriate tools for identifying ToM deficits in this neurodevelopmental pathology and establishing a more operational definition of this concept (10).

Theory of mind is a multidimensional construct that is typically defined as cognitive or affective ToM.

Cognitive ToM refers to the ability of a subject to infer epistemic states (beliefs, desires, and knowledge) devoid of emotions. As children grow, their cognitive ToM evolves, allowing increasingly elaborate thought orders generally evaluated by false-belief tasks, based on their cognitive understanding of the knowledge of a given belief (11, 12). Authors thus distinguish between the so-called 1st-order representations mastered by children at approximately 4–5 years (13) and the 2nd-order representations that evolve at approximately 6–7 years (14). According to Perner and Wimmer (15), first-order tasks entail describing the thoughts of another person concerning an event. They appeal to a child’s ability to adopt a perspective of judgment and anticipation regarding another person’s reactions, e.g., cognitive empathy. Second-order tasks involve attributing beliefs to an individual related to the thoughts of another individual. This level of understanding is grafted onto the previous one because it is based on a more elaborate level of introspection, hence its late acquisition (14, 16).

In addition, work concerning school-aged children has focused on third-order, i.e., advanced, ToM (12, 17–20)—the ability to represent a third person’s thought with one’s own thinking or to employ intellectual skills for higher processing or behavior within a social context. This order’s components manifest at school age and are grafted onto 2nd order ToM cognitive components. This high-level mentalization involves non-literal communication processes, such as irony, sarcasm, or a double bluff (7, 12). It has been called advanced ToM to emphasize its higher processing level and role in manipulating or influencing another’s thoughts or feelings, e.g., using idiomatic expressions, metaphors, allegorical proverbs, irony, sarcasm, lies, or humor (21). For all these non-literal language types, children must rely on the beliefs and intentions of the interlocutor to move beyond what is “said” and infer what is “meant.” Understanding non-literal language thus calls upon higher-level ToM skills (7) whose acquisition occurs later in development (19, 22). Thus, the ability to distinguish literal from non-literal language appears sometime between 5 and 8 years of age (6, 23). At approximately the age of five, children distinguish irony from metaphorical language (6) and lies (24); the ability to understand sarcasm and other forms of speech acts develops later (25). Such mastery continues beyond childhood and continues in adolescence and adulthood (19, 26).

Affective ToM allows accessing a representation of empathic states and therefore inferring and anticipating the feelings of others within a social context. It is necessary for interpreting the emotional valence of others’ reactions, which can be represented by actions or intentions (27–30). It is also based on the ability to recognize the emotional facial expressions that promote social interactions with others (31–34). According to authors, emotional processing in children moves through distinct developmental phases (pre-conceptual vs. intentional), which appear at an early age and continue during the school period, resulting in the development of executive control (35–37). “Detection,” i.e., the sensitivity and reactivity of young children to emotional information, is the first process established. This skill defines emotional arousal, enabling children to discriminate at least two emotional pieces of information with different valences (pleasant vs. unpleasant). Last, children interpret the intentions of others based on their expressed emotions (38). These disparate processes are fundamental prerequisites that develop gradually from birth to preschool age (27). They constitute children’s pre-conceptual level (29, 39), promoting the development of more elaborate emotional skills (40, 41) and allowing them to express themselves with a first- or even second-order thought (*A believes that B feels.*, *A believes that B believes that A feels.*, (42, 43).

Regarding the assessment of various ToM components and levels, assorted tasks and experimental paradigms are available. The most well-known are the false-belief tasks of Wimmer and Perner (44). These present stories in which a character has a belief that is different from reality (false) and the task consists of inferring the mental state of the character or her or his action based on her or his belief. These mainly conder 1st- and 2nd-order cognitive ToM capacities. According to Perner and Wimmer (15), “1st- Order False Belief” tasks aim to describe the thoughts of another person about an event. They refer to a child’s ability to adopt a perspective of judgment and anticipation for another person’s reactions, e.g., cognitive empathy. Hence, “2nd Order False Belief” tasks concern attributing beliefs to an individual based on the thoughts of another individual.

There are other paradigms for assessing the most advanced levels of cognitive ToM (e.g., “Strange Stories”) (7). These comprise a broader range of situations, including jokes, irony, sarcasm, or metaphor, for assessing different facets of high-level ToM. Similarly, tasks for assessing the pre-conceptual and elaborate levels of affective ToM have been developed. Concerning the pre-conceptual level, the detection of the intensity and valence of emotional feelings generally involves measurement scales, e.g., the “*Self-Assessment Manikin*” (27). The most elaborate levels of affective mentalization have been mainly evaluated with tests measuring children’s ability to understand and attribute emotions (28, 29).

Nevertheless, assessing the various components of ToM remains a thorny endeavor. For instance, Warnell and Redcay (45) underscore the high task dependence in any ToM task and the inability of the level of performance therein to predict performance during another task intended to measure the same underlying cognitive process. After their meta-analysis of 178 studies, Wellman and Liu (41) find that one of the limitations of most tasks for assessing ToM is how their number of items by level of difficulty increases the likelihood of random errors. In addition, as noted by Bosacki and Astington (46), even though ToM continues to develop over the whole life span periods most research and tools concern infants and preschool children (47) whereas, relevant data on the school period are scarce and more recent (18, 48–51).

Although evaluating social cognition is recommended when assessing clinical conditions (9, 10), there is still no consensus on the relevance of a specific tool for evaluating specific ToM components (52). Moreover, there are even fewer comprehensive batteries grouping evaluations of the major mechanisms at play during school-aged children’s emotional, epistemic, and advanced ToM processing of mental states (49, 53–59). Such batteries may help clinicians detect specific, detailed weaknesses in ToM processing to address observed deficits in neurotypical children or children presenting a neurodevelopmental problem. Addressing the lack of data on this developmental window, for instance, can only be achieved with tools that allow subtle assessments of school-aged children. One of the few available batteries for meeting these conceptual and methodological challenges has been proposed by Lancelot (21). The set of items integrated into this battery explicitly targets the two focal ToM components and allows the assessment of both the pre-conceptual and intentional aspects of mental states, based on the model of Coricelli (39), which demonstrates the existence of two levels of mindreading. The first level refers to the automatic–pre-conceptual phenomena that specify a basic understanding of another person’s mind and are based on early imitation, action, and emotional recognition. The second level is conceptual and voluntary, based on intentionality, empathy, and higher depths of reasoning. According to Coricelli (39), the second level of ToM development integrates both affective and cognitive components.

We have chosen to adapt the affective and cognitive evaluation battery for school-aged children by Lancelot (21) because it meets the following criteria set by Nader-Grosbois and Houssa (57) for an appropriate social-cognition assessment battery.

1. The first criterion is related to the representativeness of the ToM spanning the whole range of its dimensions. Indeed, Lancelot’s battery included a total of 10 subtests assessing pre-conceptual, affective, and cognitive aspects of ToM.
2. The second criterion refers to the presence of a variety of tasks having different levels of complexity while reducing task-dependence bias. In addition, the focal battery offers various presentation modalities with illustrations that accompany the stories told verbally and in writing.
3. The third criterion refers to various mental states that can facilitate the development of rehabilitation programs focusing on specific impaired aspects of the ToM.
4. The fourth criterion concerns the presence of highly discriminant scoring method between age groups.

Given the lack of ToM assessment tools for Arabic-speaking populations (20, 30, 34), in this study we adapt an appropriate tool and examine its validity among neurotypical Tunisian children aged 7–12 years. With the approbation of the authors, we adapted the ToM battery developed by Lancelot (21) in France to the Tunisian cultural context, specifically assessing its construct validity. Coricelli (39) and Brothers and Ring (60) have suggested that cognitive and affective components follow an ontogenetic and universal order of maturation linked to cerebral specialization. Accordingly, we hypothesize that the main ToM architectural components are universal even though ToM development involves substantial cultural variation and is sensitive to specific sociocultural type, intensity, as well as quality of social interaction. Hence, we examine whether data obtained from Tunisian School-aged children on our adapted version of the French ToM battery fit the two-component theoretical model of the theory of mind.

## Materials and methods

### Participants

Our study involving underage participants was reviewed and approved by Pr. Ahmed Khouaja, vice president of the University of Tunis, and Pr. Abdelhamid Fenina, Dean of Tunis’s faculty of humanities and social sciences, who were part of an *ad hoc* ethical committee involving human beings. The focal children’s parents provided their written informed consent for their participation in this study, and the children provided their oral informed consent.

A total of 179 Tunisian children between 83.52 months (*SD* = 0.87) and 145.53 months (*SD* = 0.65) of age participated in the study. From each of the six primary education levels, 15 girls and 15 boys (only 14 boys in the last group; sex ratio = 90/89) were randomly selected, provided they were healthy and not suffering from any mental, sensory, or motor deficits. None of the participants had learning difficulties or neurological or psychiatric disorders. All the information was obtained in semi-structured interviews with parents, teachers, and educational staff, as well as from school records (with parental authorization).

All participants were native Arabic speakers of the Tunisian dialect, and none of them were bilingual or had acquired foreign language proficiency before the age of five. The sociolinguistic particularity of the children was assessed using The Child Language Experience and Proficiency Questionnaire (61). All of them were right-handed and recruited from public educational schools in the governorate of the Greater Tunis Area, which provide the official educational programs recommended by the Tunisian Ministry of National Education. Access to schools was made possible by the approval of the ministry. The recruitment of the children took place during the 2nd and 3rd quarters of the 2020–2021 school year to ensure the adaptation of the youngest children to the school context and their familiarization with reading. Among the 200 children who agreed to take part in the research, 12 abandoned the current protocol, two were excluded because of their sensory difficulties, three were not selected due to suspicion of their mental deficiency, three had parents who were not Tunisian nationals, one was dropped due to behavioral problems, and one other was excluded because her or his scores were significantly lower than the averages for her or his age group. In total, 179 children met our criteria and completed the examinations. Table 1 lists all the sociodemographic and psychometric data for our population.

**TABLE 1:** Socio-demographic and psychometric data.

Education level	1st year (*n* = 30)	2nd year (*n* = 30)	3rd year (*n* = 30)	4th year (*n* = 30)	5th year (*n* = 30)	6th year (*n* = 29)	Total (*n* = 179)
**M (SD)**							
Age (months)	83.52 (0.87)	96.23 (0.71)	109.8 (0.84)	118.63 (0.74)	133.45 (0.89)	145.33 (0.65)	114.49 (0.78)
Mother’s education (years)	10.35 (0.66)	10.20 (0.53)	10.4 (0.63)	9.13 (0.56)	9.27 (0.67)	9.81 (0.49)	9.86 (0.59)
Father’s education (years)	9.96 (0.55)	10.6 (0.45)	9.32 (0.53)	9.75 (0.47)	8.95 (0.57)	9.05 (0.41)	9.60 (0.50)
Raven matrices PM47 (raw score)	17.95 (0.78)	17.97 (0.63)	19.56 (0.74)	21 (0.66)	21.68 (0.79)	26 (0.57)	26.69 (0.69)

As shown in Table 1, the different age groups are comparable in terms of the number of years of education of the father [*F*_(5, 173)_ = 1.17; *p* = 0.121; *ηρ*^2^ = 0.05] and the mother [*F*_(5, 173)_ = 0.85; *p* = 0.519; *ηρ*^2^ = 0.02]. The groups also demonstrate similarly fluid intelligence [*t*_(177)_ = 0.19; *p* = 0.847] among boys and girls in the same age group, with an improvement in average scores between 7 and 12 years [*F*_(5, 167)_ = 22.82; *p* < 0.0001; *ηρ*^2^ = 0.41]. Similarly, we observed a non-significant effect of interaction between age and sex on variation in the level of fluid intelligence [*F*_(5, 167)_ = 0.16; *p* = 0.976; *ηρ*^2^ = 0.01].

### Material

#### The ToM battery

The focal battery was constructed by Lancelot in the French language (21, 62). Lancelot stated that the selection of the items and subtests during the designing process of the battery was theoretically rooted in the clinical and developmental seminal works of Baron-Cohen et al. (63), Brothers and Ring (60), Coricelli (39), and Wellman (29). The battery is composed of three components:

1) A pre-conceptual component containing two subtests: (i) estimation of arousal and valence, and (ii) facial recognition of emotional expressions in drawings and photos.
2) an affective component containing three subtests: (i) Attribution of emotions according to social context, (ii) distinction of apparent emotions/real appearances, and (iii) detection of a social misstep.
3) A cognitive component containing two subtests: (i) First- and second-order false beliefs, and (ii) advanced cognitive ToM.

##### The pre-conceptual and emotion recognition part of the ToM battery

The pre-conceptual part of the battery was conceived as a control stage prior to the assessment of mental states. It was not calculated in the analysis. Rather, it only allows certainty of the integrity of two basic aspects: (a) The capacity to detect the intensity (arousal) and valence of an emotional feeling; (b) the capacity to recognize facial emotional expressions in drawings and photos.

###### Detection of the intensity (arousal) and valence of an emotional feeling

The child is shown 30 photographs and is asked to indicate on the “Self-Assessment Manikin” cards (27) the valence (negative, positive, and neutral) and intensity (low, medium, and high) of an emotional feeling experienced for each photograph.

###### Recognition of emotional facial expressions (drawings and photos)

The test is carried out in two stages. In the first stage, we administer a task involving the recognition of drawings’ emotional facial expressions; we present 10 boards with hand-drawn faces, five of which correspond to boys’ faces and five to girls’, displaying a characteristic emotion of joy, fear, anger, sadness or, finally, surprise. The child is asked to identify the emotion on each face. In the second stage, we employ the task of recognizing photos’ emotional facial expressions (64); we present 12 boards with photos of the faces of men and women displaying a characteristic and basic emotion. The emotions, represented by 2 items each, are joy, fear, anger, sadness, surprise, or disgust.

##### The affective component of the ToM battery

This part is composed of three tests: attribution of emotions according to social context; detection of social missteps (faux pas); and distinction between “apparent vs. real emotion.”

###### Attribution of emotions according to social context

This contains 10 different situation stories, presented in comic strips. Each relates to the adventures of the characters. At the end of each comic strip, the protagonist’s facial expression is deliberately absent. The child is thus asked to choose the most appropriate emotion for the feelings of the protagonist, confronted with the evoked social situation with pieces of cardboard representing faces with basic emotional expressions relating to joy, fear, sadness, anger, or surprise.

###### Detection of a social misstep

These are eight verbally communicated stories in which an individual expresses social awkwardness about another character (5). In its cognitive section, the child must identify the misstep and justify the cause. In its affective section, the child must attribute to the victim an emotional valence in line with the offense felt by the character. Four control situations “without missteps” are integrated to ensure the mastery of this skill by the child.

###### Distinction between “apparent or real emotion”

This subtest includes two stories where the child is confronted with two vexing situations for the protagonist. To hide her or his vexation, the real emotional feelings are replaced by a so-called apparent emotion that contrasts with them. The child must discriminate the real emotional feeling of the character from her or his appearance by responding to four questions. The first question refers to the perception of the apparent emotion (perception question). The second refers to the child’s belief regarding the perception; the child must justify her or his answer. The third question entails the categorization of the felt emotion, followed by a fourth question where the child must justify her or his answer.

##### The cognitive component of the ToM battery

This part includes two subtests evaluating 1st- and 2nd-order false-belief tasks taken from Perner and Wimmer (15) and ten strange stories from Happé (7) representing the three dimensions described below.

###### First- and second-order false-belief tasks

These include adapted scenarios of “Sally and Ann” (63) and “Max and Chocolate” (14, 15). Each of the different stories is performed in a concrete way by moving two doll characters and objects. Providing the child with two stories assessing the same mental function not only controls for administration bias but also, *via* character and setting changes, ensures the child has mastered the tasks’ 1st-order false beliefs.

In the 2nd-order false belief tasks, an absent character who is being tricked watches the action performed by the second character from a hidden location (looking out a window without the other character’s knowledge). These are so-called 2nd-order false-belief stories from the adapted version of Sally and Ann (14), supplemented by the story of “Chloe’s cakes” (14).

###### The cognitive aspects of advanced ToM

This part of the battery comprises ten different tasks where various advanced ToM aspects are investigated. The intellectual work that the child must perform is based on the understanding of the mechanisms of empathy, imagination, language proficiency, and cultural and social knowledge. The humor detection task is based on eight short stories derived from experimental tests (22, 65); the child is invited to assess their humorous content. The other tasks are adaptations of Happé’s “Strange Stories” (7), e.g., “pretending,” “appearance/reality distinction,” “persuasion,” “joke,” “misunderstanding,” “double bluff,” “metaphor,” “lying,” and “sarcasm.” The advanced cognitive component of the ToM battery thus assesses three dimensions: “manipulation” (sarcasm, double bluff, and persuasion), “disguise” (lying, misunderstanding, apparent reality, and joke), and “substitution” (humor, pretending, and metaphor).^1^

The parents of the children received a brief written presentation of the research and a consent form specifying that the information provided and the results of the testing would be confidential and anonymous, only be used for research purposes. After signing the consent form, they completed a demographic information sheet.

All the tests were individually administered in a calm place without any distractions and had an average duration of 2 h, spanning two sessions. Breaks were granted as needed. The cognitive and affective ToM subtests were counterbalanced, i.e., two items from the same subtest were not presented successively. The stories told to the children could be accompanied by a visual medium (comics, photographs, images, or even written texts) or performed using dolls and material specific to the focal situations (cardboard house, Playmobil, confectionery, etc.).

Notably, all the subtests and instructions were provided in the Tunisian dialect to promote better task understanding (66, 67).

## Results

Data can be accessed *via* this OSF link: https://mfr.osf.io/render?url=https://osf.io/nu6fx/?direct%26mode=render%26action=download%2358 6mode = render.

### The child language experience and proficiency questionnaire analysis

All the children in our sample have mastered primarily the Tunisian dialect that was followed by standard Arabic, then French, and lastly some elements of English, both in terms of dominance and acquisition. The average exposure time of the dialect is 100 and 95% for Standard Arabic, 80% for French and 48% for English. When it comes to the possibility of reading a text, 83% prefer it to be in standard Arabic and 32% in French. No one searches preferentially in English when all language versions are available. In terms of the child’s proficiency in speaking and verbal comprehension, the parents stated that the proficiency follows this order: Tunisian dialect, standard Arabic, French, and finally English while in reading and writing the order is as follows: standard Arabic, French, and English. The parents think that their children are better in French than standard Arabic for 21% of the cases, and than English for 2% of the cases. In addition, the parents think that the factors with the most significant impact on their child’s linguistic status were ordered as follows: Interactions with family, interactions with friends, reading, self-learning *via* Internet, browsing and video games, then movies and television. This is consistent with the degree of exposure to these contexts and channels of communication.

### The translation and adaptation of the ToM battery

It is crucial to note two important details: first, the fact that the choice of a French tool was strongly motivated by the geographical, historical, social proximity between France and Tunisia. In fact, previous studies on the validation of psychometric batteries from French to Tunisian culture, showed similar patterns of performance (68, 69). Second, the fact that battery translation and adaptation were performed after the approval of Lancelot.

As far as the translation and adaptation processes are concerned, we note that we adhered to the following steps recommended by the International Test Commission (70): (a) The first stage consisted of a translation (“Translation”) of the material (the content of the stories/comprehension questions and target questions) of the cognitive and affective ToM from the French language (original version) to the Arabic Tunisian dialect (commonly used in conversations in daily life and with which Tunisian children are very familiar). Two bilingual expert teacher–researcher psychologists completed this translation. (Translator 1: YH; Translator 2: SB), which was based on the cultural, linguistic, and contextual characteristics of Tunisian society while respecting the psychometric particularities of the battery (71). The second step (b) entailed merging the two translated versions, which was carried out by two other psycholinguist teacher–researcher experts (Translator 1: RBR; Translator 2: AN) and allowed evaluating their equivalence in terms of semantics and concepts to verify the contextual and idiomatic aspects. This step was followed by (c) verifying the correct understanding of the scenarios, the handover instructions, the comprehension questions, and the target questions as well as the scoring procedures. Following Borsa, Damasio, and Bandeira (72), in the fourth step (d), we evaluated the material directly with an exploratory sample composed of 45 Tunisian children aged 7–12 years. Overall, the content of the battery did not pose major problems for understanding; nevertheless, we had to make some qualitative readjustments (improvement in the quality of the drawings, modification of some concepts that could be applied to all the specificities of the regional dialect(s), adding two scenarios for the evaluation of persuasion based on one component of advanced ToM). The last step entailed (e) back-translation into the French language, which was performed by two novel bilingual experts (Translator 1: TB; Translator 2: SBB). This last phase allowed us to adjust the quality of the translated tool by matching the initial version to the version that had undergone “translation–back-translation.” Interrater agreement was very high (Kappa = 0.98). Our final verification of the material occurred through our study with the focal population (73).

At the end of the “translation–back-translation” procedure, the scores and percentages were calculated by comparing the original French version with the back-translated Arabic Tunisian version across all 10 subtests and all statements in the original version. We found that the terms are similar when they are synonyms. The similarity rate is estimated at 100%.

### The validity of the battery

The construct validity of the Tunisian version of the ToM battery was tested using structural equation modeling (SEM). The predictive validity was tested by examining the effects of age on the development of cognitive and affective ToM using mixed ANOVAs followed by Newman–Keuls *post hoc* analyses.

### Construct validity

**FIGURE 1:**
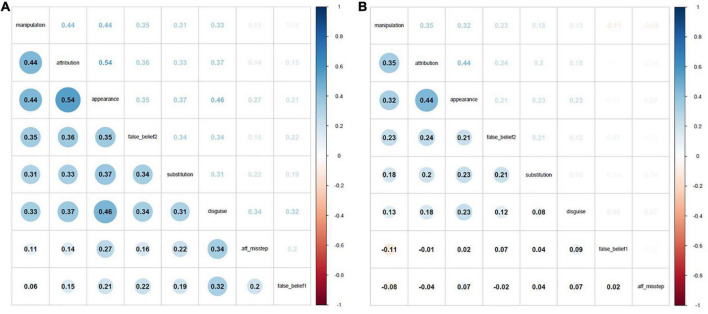
Correlograms between the standardized ToM scores before **(A)** and after controlling for age **(B)**. Heatmap values highlight negative correlations in red and positive in blue. Note that the size of the circles and the shading color of the correlation values are a function of the strength of correlations. The variables were ordered based on the angular order of the eigenvectors.

Construct validity was assessed using SEM. The two latent variables, cognitive and affective components, are reflected in the following ToM scores: *attribution of emotions according to social context* (*attribution emotion), distinction between “apparent* and *real emotion” (appearance/reality), and detection of a social misstep (affective misstep)* for the affective dimension; *1st- and 2nd-order false-belief tasks* (*false belief 1 and 2);* and three dimensions of advanced ToM—*substitution, disguise, and manipulation for the cognitive dimension*. Due to a clear effect of age on ToM score, we adopted SEM with a correlation matrix based on standardized scores after controlling for the age variable, as shown in Figure 1. Such methodological caution allows assessing the construct validity of the battery independently of age variation in performance.

**FIGURE 2:**
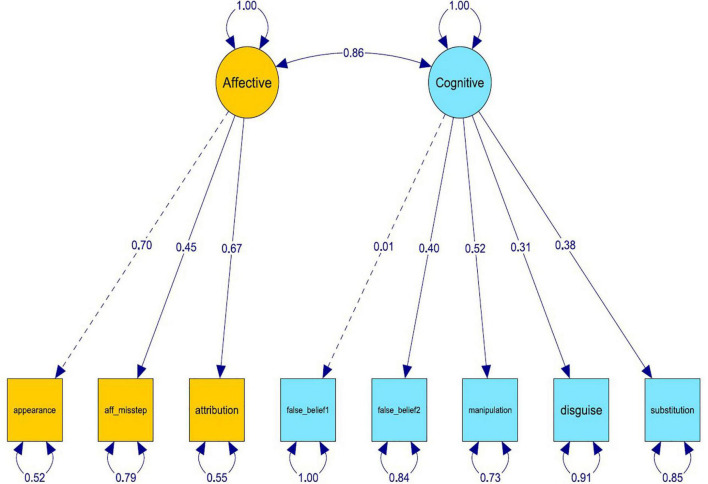
Structural equation modeling model with standardized structural coefficient estimates of the relationship between each latent variable and the corresponding exogenous variables.

Structural equation modeling was performed using the R package lavaan (74). Assuming that the data collected are continuous, the estimation of the model parameters used maximum likelihood. These results revealed that the goodness-of-fit indices suggest an adequate fit of the data with the hypothesized model. Overall, this chosen bipartite model is reasonably consistent with the data (*Chi-square* = 15.52, *df* = 19, *p* = 0.689). Moreover, the comparative fit index (*CFI* = 1.00) is higher than the cutoff value of 0.90, while the values of the standardized root mean square (*SRMR* = 0.041) and root mean square error of approximation (*RMSEA* = 0.000) are lower than the cutoff value of 0.08, entailing a good fit. Figure 2 illustrates the fitted model.

### Predictive validity: Examining the age effect

To verify the two ToM components validated by SEM analysis support this prediction and to show the presence of differential developmental trajectories between cognitive and affective components, we performed mixed analysis of variance with age (9, 17, 75–78) as a between-participants factor and component (cognitive vs. affective) as the within-participants factor on the variation in the total relative score across the ToM components. These results revealed a significant effect of age [*F*_(5, 173)_ = 34.79; *p* < 0.001; *ηρ*^2^ = 0.50], suggesting that age significantly affects performance on ToM relative scores; a significant component effect [*F*_(1, 173)_ = 350.77; *p* < 0.001; *ηρ*^2^ = 0.67], suggesting differential developmental trajectories between cognitive and affective components; and a significant interaction [*F*_(10, 346)_ = 2.91; *p* < 0.05; *ηρ*^2^ = 0.08). The latter was subjected to a Newman–Keuls *post-hoc* test.

**FIGURE 3:**
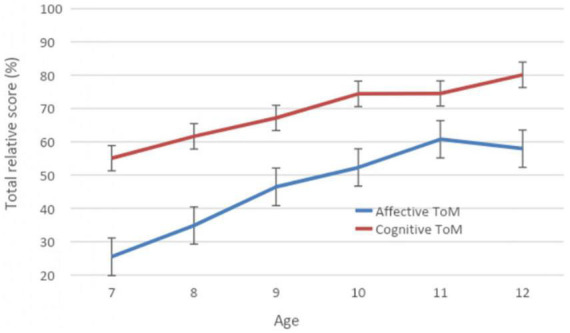
Variation of the cognitive and affective total relative scores at the ToM battery according to age.

These results show that across all the focal age groups, the children succeeded more in cognitive tasks than affective tasks (*p* < 0.001). Concerning the developmental trajectory of each component (Figure 3), the results show, for the affective component, significant differences regarding the total relative scores between children aged 7 and 8 (*p* < 0.01), between children aged 8 and 9 (*p* < 0.005), and among children aged 9 and 11 or higher (*p* < 0.005). No significant differences were observed among children aged 10, 11, and 12. For the cognitive component, the developmental trajectory is quite different from the affective one, with no significant difference in the total relative scores between children aged 7 and 8 (*p* = 0.227) but there were such differences among children aged 7 and 9 or higher (*p* < 0.005). There was no significant difference in the total relative scores between children aged 8 and 9 (*p* = 0.110) but there were such differences among children aged 8 and 10 or higher (*p* < 0.005). Unlike the affective component, the difference in the total relative scores between children aged 9 and 10 is significant (*p* < 0.05). However, similarity to the developmental trajectory of the cognitive and affective components was observed among children aged 10, 11, and 12, even though the cognitive relative scores were higher than the affective relative scores.

Overall, then, we have demonstrated two major findings: First, there are significant differences between the cognitive and affective levels of performance across all age groups, in favor of the cognitive component. This component effect also strengthens the dissociation between the two ToM components. Second, we have noted continuous improvement in the performance of children aged 7, 8, and 9 years, with distinct developmental trajectories for the cognitive and affective components. However, we observe no significant differences in ToM performance in both components for ages 10, 11, or 12.

## Discussion

The main objective of this study was to examine the construct validity of an adapted French version of the ToM battery for Tunisian school-aged children. One of the few available batteries that could be an effective candidate for a comprehensive and subtle assessment of ToM components [see Coricelli (39)] is that of Lancelot (21).

Our findings were obtained using covariate scores. Our procedure was motivated by the non-linear age effect on disparate ToM components and the significant interaction observed between age and each ToM component.

Our results, stemming from data after controlling for age, confirm the construct validity of the adapted version of the battery *via* SEM, showing that the two-component model of ToM (cognitive and affective components) fit the data very well. Moreover, these results confirm the widely accepted assumption that ToM is a non-unitary construct for clinical (79–82), neuroimaging (17, 83), and developmental data (49, 58, 84–86).

Indeed, the dichotomous structure of ToM has been observed using clinical data. For example, Baldimtsi et al. (82) demonstrate that the performance of children with ASD is selectively impaired in both ToM aspects, supporting the distinction between them. Tager-Flusberg and Sullivan (81) similarly reveal this disfavor of affective ToM in their study on children with Williams syndrome. Results in favor of the existence of the two components of ToM have also been demonstrated in a population with high and low extents of somatic symptoms (87) and in alcohol-dependent patients (88).

On the other hand, the validation of the two ToM components affirms the results in Kalbe et al.’s (89) neuroimaging study of healthy male participants performing cognitive and affective computerized ToM tasks. These results show that repetitive transcranial magnetic stimulation (rTMS) on the right dorsolateral prefrontal cortex (DLPFC) selectively affects cognitive but not affective ToM tasks. These authors interpret these results as proof of the functional independence of cognitive and affective ToM.

Our results also align with developmental studies such as Meinhardt-Injac et al. (84), who validate the two-component model (social perceptual and social-cognitive) among adolescents and young adults *via* the asymmetries in their development trajectories using linear mixed models (LMMs). The social perceptual component shows specific growth, while the social-cognitive component is aligned with the development of language, reasoning, and inhibitory control. Our study validates this two-component model with SEM analysis while covarying age. Therefore, our results are in line with previous studies defending the two-component approach to ToM (39, 81, 89). For example, Coricelli (39) has identified two levels of mindreading. The first level refers to the automatic–pre-conceptual phenomena that specify a basic understanding of another person’s mind. It is based on early imitation, action, and emotional recognition. The second level of mindreading is conceptual and voluntary, based on intentionality, empathy, and higher depths of reasoning. According to this author, the second level of ToM development integrates both affective and cognitive components. With age development and amid neuronal and social maturation, children become able to process more complex representations, including conative pragmatic ToM (17). These processes seem to share common mechanisms with those identified in our factor analysis, which are related to manipulation, disguise, and substitution. Thus, at school age, children have to demonstrate social reasoning (substitution), reasoning about ambiguity (disguise), and the recognition of social norm transgressions (manipulation).

Furthermore, our findings based on the analysis of the effect of age on different ToM components reaffirm validity of the two-component battery model by showing a significant component effect and distinct trajectories for the two components. This confirms previous studies that have revealed various behavioral expressions of ToM in different age groups (90, 91), reinforces the dissociation between the affective and cognitive components, and strengthens the validity of this battery. These results also highlight the developmental trends of each of the two components among 7–12-year-old Tunisian children, confirming previous studies showing an increase in ToM scores with age in typically developing children (54, 55).

Furthermore, our results show that in regard to the variation in the average success rates noted in the tasks evaluating affective ToM, these register performances significantly lower than those for cognitive ToM, with a plateau of performance from the age of 10 years. In other words, school-aged Tunisian children reach performance stability at an earlier age than for the cognitive dimension. On the other hand, affective ToM performance develops rapidly between 7 and 9 years and then slows. This is concordant with Dennis et al. (17), who have found that among children with brain damage aged between 8 and 13 years, affective and high-level cognitive ToM processing has a lower disturbance threshold than 1st- and 2nd-order cognitive ToM (even during moderate CBT). These authors demonstrate the more significant impairment of these last two ToM components by the mobilization of denser neural circuits (mirror neuron empathy, central executive, and default mode networks) than those mobilized during 1st- and 2nd-order cognitive tasks. These circuits undergo a longer maturation, given the increased number of neural networks solicited by affective and high-level/advanced cognitive components. This explains their later onset than 1st- and 2nd-order cognitive ToM and their greater vulnerability to brain damage at school age. This argument therefore favors splitting ToM into different components. Similarly, when comparing performance on affective and advanced cognitive tasks, the above authors find that the accuracy of responses to advanced ToM tasks was significantly higher than affective ToM tasks. These differences in performance in favor of advanced cognitive ToM are in line with our results, which highlight stability in the performance of affective ToM from the age of 10 years. These data mirror Bialecka-Pikul et al. (92) and Shamay-Tsoory et al. (93), who suggest that affective ToM is based on complex processing *via* empathic behaviors while integrating the mechanisms necessary for carrying out cognitive mentalization. Some of these processes can only emerge from social interactions that peak only in adolescence, hence their stagnation during the school period. Conversely, advanced cognitive ToM is dependent on the recursive language processing capacities that develop significantly during the school period.

### Limitations

Some important issues need to be addressed. The first is the conceptualization of ToM, especially its advanced aspects. Across studies, the definition and operationalization of affective and cognitive ToM are fairly stable, but for advanced ToM, they are inconsistent, with important variability in the tasks adopted for studying it. For example, advanced ToM has been defined and measured as the ability to interpret emotional cues in pictures of eyes in some studies (94, 95) or as the ability to reason about others’ thoughts, feelings, and behaviors in response to social stories in others (83, 84). Our focal ToM battery (21), adapted to and validated *via* testing in Tunisia, integrates ten tasks selected from previous experiments (22, 65) and Happé’s “Strange Stories” (7). These data, issued from the administration of these tasks, were subjected to PCA, which yielded three factors—“manipulation” (sarcasm, double bluff, and persuasion), “disguise” (lying, misunderstanding, apparent reality, and joke), and “substitution” (humor, pretending, and metaphor). This entails a conative high level of mentalization, using, voluntarily, the capacity to represent a thought that a third person makes of our own thought in a social context. In addition, using multiple tasks to attenuate the test effects in Lancelot (21) is appropriate; most studies have adopted a single measure to assess advanced ToM (94). Warnell and Redcay (45) point out the high task dependence of ToM tasks and that the level of performance in one ToM task cannot predict performance in another task assumed to measure the same underlying cognitive process. In their meta-analysis of 178 studies, Wellman and Liu (41) find that one of the limitations of most tasks adopted to assess ToM is that the number of items, by level of difficulty, increases the chances for random errors.

The second issue is the status of the pre-conceptual ToM tasks in the ToM battery (21). As noted above, the pre-conceptual part of the battery was integrated following Decety’s model (96) to examine the integrity of two basic pre-requisites for intentional affective ToM, i.e., the capacity to detect the intensity (arousal) and valence of an emotional feeling and the capacity to recognize facial emotional expressions. The scores for these pre-conceptual aspects serve as methodological controls and have clinical implications. They should be acquired earlier in development, between 2 and 5 years of age (96) and are thus not integrated into the total affective ToM scores.

Third, we adopted only SEM and age predictive validity to test the construct validity of the translated adapted version of the ToM battery. Among the different methods for test validation, we did not select face validity due to its weakness (97). For the criterion validity options, we could not perform concurrent validity since we lacked any other tools in Arabic to use as a parallel form. Meanwhile, predictive clinical validity could be an excellent option but will have to be applied in a follow-up project. Construct validity offers strong evidence for the validity of measurement. It allows, for instance, SEM to verify its nomological network by confirming that the gathered data fit the theory predictions. To our knowledge, no study in the ToM literature has addressed the construct validity of a two-component model using SEM. Even though inaccurate definitions of the construct can be considered its major threat, the construction of the focal battery was, as mentioned above, theoretically grounded, and our use of multiple well-known subtests significantly reduced this risk. This approach also prevents any mono-operation bias (98). Finally, the use of age as a variable for follow-up predictive validity was motivated by the attempt to revalidate the two-component ToM model confirmed by SEM analysis. This step has offered additional evidence for the distinction between cognitive and affective ToM.

Fourth, the cultural dimension of ToM processing was not analyzed, as this was not the main objective of this psychometric article. Our translation–adaptation procedure followed the best practices suggested by the recommendations of the International Test Commission (70) and signaled that the main architectural component remains universal, even though the developmental trajectories are sensitive to cultural variation. Since the original battery was written in French, we plan a cross-cultural study comparing Tunisian and French data. Such a study will shed light on the similarities and differences in ToM processing according to culture. Anh and Miller (99) have found that Korean children who exhibit more collectivist self-concepts perform better on false beliefs than American children, whereas Oberle (100) and Oh and Lewis (101) were unable to note any differences in ToM performance between children from individualistic and collectivistic cultures. Finally, we are not aware of any study showing that in some cultures, ToM is unitary or has more than two components (cognitive and affective).

## Conclusion

Although the assessment of social cognition is recommended in clinical situations (9), there is still no consensus on the relevance of a specific tool for examining particular ToM components (53). Very few comprehensive batteries include an evaluation of the significant mechanisms at play during emotional, epistemic, and high-level cognitive processing of mental states in school-aged children (48, 54–59). However, Tunisian practitioners and researchers lack any linguistically and culturally appropriate psychometric tool for ToM assessment in school-aged typical and atypical children. Therefore, our study has assessed the construct validity of a translated and adapted French ToM Battery among Arabic-speaking Tunisian school-aged children. This battery was designed to allow the investigation of (a) cognitive ToM abilities with 1st- and 2nd-order scenarios, explaining false-belief challenges and high-level/advanced components (manipulation, disguise, and substitution); and (b) affective ToM with measures of inferences of emotions according to social context, distinction between apparent/real emotions, and recognition of emotional missteps. Ultimately, we have provided a systematic evaluation of ToM in children in clinical and research settings; thus, our findings have implications for the optimal management strategies for patients, specifically school-aged children on the autism spectrum (21, 67).

The aim of this study was to examine the construct validity of the focal battery, one of its major psychometric qualities, using SEM. Our results mirror the conceptual models supported in previous studies (39) and confirm the existence of the two components of ToM. However, additional validity data *via* clinical and non-clinical samples are needed. Data on cognitive and affective ToM evaluation battery in children indicate a gradual and continuous improvement in the performance of children aged 7–12 years concerning the intentional decoding of affective and cognitive components of mentalization. Such variation during this developmental period reflects the literature and affirms the sensitivity of our tool to the Tunisian population (17, 21, 39, 60, 63, 67). These data are thus demonstrating the effective differential validity of the content of our battery.

Finally, our data, obtained from a sample of school-aged Tunisian children using a Tunisian version of the ToM battery, confirm its validity and allow its use in both clinical and developmental research, especially among children with neurodevelopmental disorders. The question regarding whether the instrument can be extended to all Arabic-speaking children is legitimate. Although the test is translated and administrated using the Tunisian Arabic dialect, this does not exclude the fact that it can be used in other Arabic countries with different dialects. Therefore, a follow up studies can alleviate such issue by providing a version with standard Arabic that can be understood univocally across all Arabic speaking countries.

## Data availability statement

The original contributions presented in this study are included in the article/supplementary material, further inquiries can be directed to the corresponding author.

## Ethics statement

The studies involving human participants were reviewed and approved by Pr. Ahmed Khouaja, Vice-President of the University of Tunis, and Pr. Abdelhamid Fenina, Dean of Tunis’s Faculty of Humanities and Social Sciences, members of the ethics committee. Written informed consent was obtained from the participants’ legal guardian for the publication of any potentially identifiable images or data included in this article.

## Author contributions

IS: data collection, data analysis, statistical analyses, and manuscript writing. CL: study design, construction tools, and design the procedure for social recording. YM: statistical analyses and manuscript writing. WS and AB: data collection and manuscript writing. DL: manuscript writing. TB: data analysis, statistical analyses, and manuscript writing. All authors contributed to the article and approved the submitted version.

## Footnotes

1 Principal component factor analysis (PCFA) with varimax rotation was performed on the relative scores of the 10 advanced ToM tasks: Humor, pretending, appearance/reality distinction, persuasion, joke, misunderstanding, double bluff, metaphor, lying, and sarcasm. PCFA results yielded a 3-factor solution explaining 46.85% of the total variance—16.54, 16.10, and 14.21%, respectively. These factors can be labeled as (a) “manipulation,” composed of scores on sarcasm, double-bluff, and persuasion; (b) “disguise,” defined by high loadings for lie, misunderstanding, apparent-reality, and joke tasks; and (c) “substitution,” based on the scores for humor, pretending, and metaphor.
